# Application of RF-MEMS-Based Split Ring Resonators (SRRs) to the Implementation of Reconfigurable Stopband Filters: A Review

**DOI:** 10.3390/s141222848

**Published:** 2014-12-02

**Authors:** Ferran Martín, Jordi Bonache

**Affiliations:** CIMITEC, Departament d'Enginyeria Electrònica, Universitat Autònoma de Barcelona, Bellaterra (Barcelona) 08193, Spain; E-Mail: jordi.bonache@uab.cat

**Keywords:** RF-MEMS, split ring resonators (SRRs), metamaterials, stopband filters, reconfigurable components

## Abstract

In this review paper, several strategies for the implementation of reconfigurable split ring resonators (SRRs) based on RF-MEMS switches are presented. Essentially three types of RF-MEMS combined with split rings are considered: (i) bridge-type RF-MEMS on top of complementary split ring resonators CSRRs; (ii) cantilever-type RF-MEMS on top of SRRs; and (iii) cantilever-type RF-MEMS integrated with SRRs (or RF-MEMS SRRs). Advantages and limitations of these different configurations from the point of view of their potential applications for reconfigurable stopband filter design are discussed, and several prototype devices are presented.

## 1. Introduction

**Figure 1.**
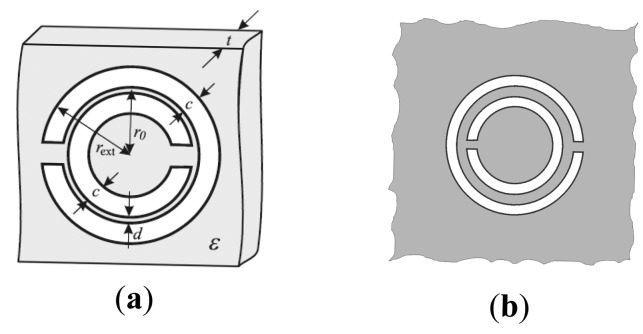
Typical topology of a metallic split ring resonator (SRR) (**a**), and complementary SRR (CSRR) (**b**). The relevant dimensions are indicated in (a).

Split ring resonators (SRRs) [1] and their complementary counterparts, *i.e.*, complementary split ring resonators (CSRRs) [2], are key building blocks for the implementation of metamaterials [3–5] and metamaterial transmission lines [6,7]. SRRs (see the typical topology in Figure 1a) are electrically small resonators by virtue of the electric coupling between the inner and outer ring. These resonant elements can be excited by means of an axial time-varying magnetic field, or by means of an electric field applied in the plane of the particle, in the direction orthogonal to its symmetry plane. Due to its small electrical size, SRRs can be used for the implementation of effective media metamaterials [3,5]. Specifically, it has been demonstrated that an array of SRRs illuminated by an incident electromagnetic radiation polarized with the magnetic field axial to the rings behaves as a negative permeability medium in a narrow band above the SRR fundamental resonance [3]. By loading a transmission line with SRRs, a one-dimensional negative permeability metamaterial also results [6]. Due to the negative effective permeability above the SRR fundamental resonance and to the high positive permeability below it, a stop band in the vicinity of that frequency results, and the structure exhibits stopband functionality.

It has been demonstrated from duality arguments that CSRRs (Figure 1b) can be applied to the design of negative permittivity one-dimensional metamaterials (or metamaterial transmission lines) [7,8]. CSRRs can be either excited by means of an axial time varying electric field, or by means of a magnetic field applied in the plane of the particle (orthogonal to its symmetry plane). By loading a transmission line with CSRRs, a stop band in the vicinity of the CSRR fundamental resonance also results, but in this case the filtering action is related to the extreme values (positive/negative) of the effective permittivity.

**Figure 2.**
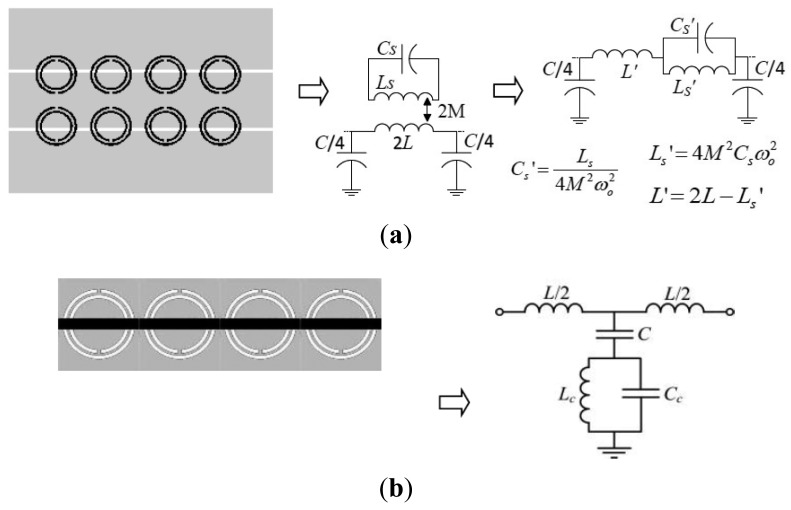
Typical layout and circuit model (unit cell) of an SRR-loaded line (**a**) and CSRR-loaded line (**b**). In (a), the magnetic wall concept is applied. *L* and *C* represent the per-section transmission line inductance and capacitance, respectively, *C_s_-L_s_* and *C_c_-L_c_* are the capacitance-inductance of the SRR and CSRR, respectively and *M* is the mutual inductance between the SRRs and the line. In (a), the circuit model can be transformed to the one in the right hand side, with the indicated transformations.

The stopband functionality of SRR- or CSRR-loaded lines can also be interpreted from the lumped element equivalent circuit model of the unit cell of these lines, both depicted in Figure 2. These models are valid as long as the resonators are electrically small, *i.e.*, up to frequencies slightly above the fundamental resonance [7]. Note that for the model of Figure 2a, a transmission zero at the intrinsic resonance frequency of the SRR arises:

(1) $$\omega_{z}=\frac{1}{\sqrt{C_{s}L_{s}}}=\frac{1}{\sqrt{C{'}_{s}L{'}_{s}}}$$

whereas for the structure of Figure 2b, the transmission zero is given by:

(2) $$\omega_{z}=\frac{1}{\sqrt{(C+C_{c})L_{c}}}$$

If the distance between adjacent resonators is small, inter-resonator coupling may be important. If this is the case, the stop band bandwidth is enhanced due to the appearance of complex modes, as it has been demonstrated in [9,10], and the circuit model of the unit cell is a multi-terminal network. Another strategy for bandwidth enhancement is to consider multiple tuned structures, namely, SRRs, or CSRRs, tuned at slightly different resonance frequencies within the desired stop band [11]. This sacrifices periodicity, but it has been demonstrated to be an efficient approach in order to control the stopband bandwidth.

Several strategies/technologies for the implementation of reconfigurable/tunable stopband filters based on SRR- or CSRR-loaded lines have been reported. Among them, loading the resonators with varactor diodes [12–14] is a possibility, but the operation frequency of these elements is limited. SRRs or CSRRs can also be etched on top of tunable materials, such as ferroelectrics [15,16]. This strategy provides tunability, but the required tuning voltages are typically high, and losses may degrade filter performance. The combination of RF-MEMS with SRRs, or CSRRs, is an alternative approach that has been demonstrated to provide very reasonable performance and high frequency operation [17]. This review paper is focused on this later tuning approach. We will consider different specific strategies, in particular, split rings with RF-MEMS loading (including bridge and cantilever type RF-MEMS), where tuning is achieved through electrostatic actuation on a set of MEMS switches loading the resonators [17–21], and cantilever-type SRRs, where the arms of the SRR are deflectable [22]. The ability of RF-MEMS technology to provide an efficient solution to the tuning of microwave circuits has been demonstrated over the past years [23–27], and RF-MEMS have also been applied to the implementation of SRR-based tunable metamaterials [28,29]. In this work, it is demonstrated that there are several alternative configurations of SRR/RF-MEMS combinations useful for the implementation of metamaterial-inspired tunable stopband filters.

## 2. Stopband Filters Based on Split Rings with RF-MEMS Loading

Let us first consider the implementation of reconfigurable stopband filters based on SRRs and CSRRs with RF-MEMS loading. SRRs have been loaded with cantilever-type SRRs and applied to the implementation of stopband filters in microstrip technology [21], whereas CSRRs combined with bridge type RF-MEMS have been demonstrated to be useful reconfigurable resonators for the design of stopband filters in coplanar waveguide (CPW) technology [17–20]. Let us now review the two approaches separately.

### 2.1. CSRRs Loaded with Bridge Type RF-MEMS

**Figure 3.**
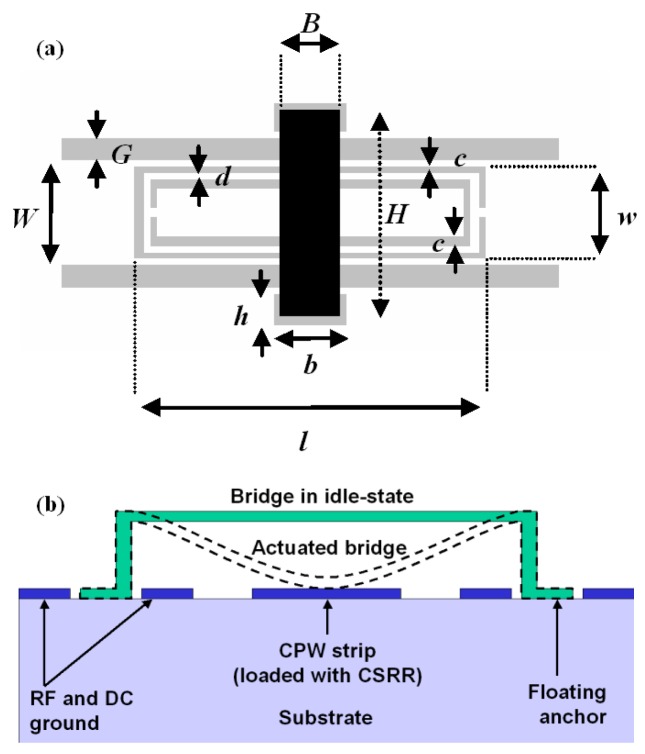
(**a**) Unit cell of the CSRR/RF-MEMS loaded coplanar waveguide (CPW), with slot regions of the CPW depicted in grey, and relevant dimensions; (**b**) cross section of a CPW with a RF-MEMS bridge. The down-state corresponds to the application of an actuation voltage to the strip line of the CPW; in the up-state, no actuation voltage is applied. From [17], reprinted with permission.

The first reviewed stopband filters are implemented in CPW technology by means of tunable CSRRs using fixed-fixed beam RF-MEMS [17–20]. The CSRRs are etched in the central strip of the CPW, and the RF-MEMS are implemented on top of them, as Figure 3 illustrates. The RF-MEMS structures use an electrostatic floating bridge anchored on the substrate in holes of the CPW ground planes. Through electronic actuation the MEMS are bended down, modifying the effective capacitance of the CSRRs and hence the resonance frequency.

**Figure 4.**
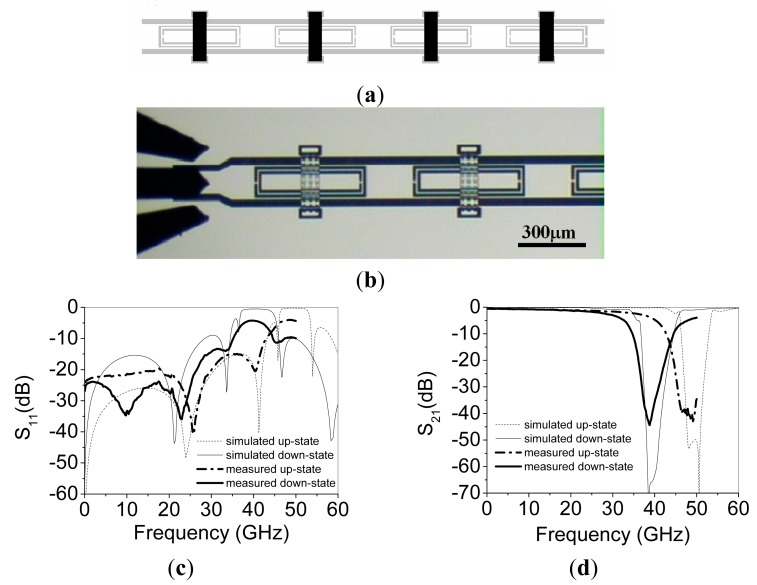
Layout of the fabricated tunable stopband filter (**a**), microphotograph of the first two stages of the filter, including RF probes (**b**), simulated and measured insertion losses (**c**), and simulated and measured return losses (**d**). The simulations were done by considering plate heights of 0.5 μm and 2 μm for the down and up-state, respectively. From [17], reprinted with permission.

A stripped-down RF-MEMS technology using only 3 lithographic steps [17] to define the structures of Figure 3 is used. First, a 1-μm thick Al layer is sputter-deposited and patterned on a 650-μm thick AF45 glass substrate (*ε_r_* = 5.9) to define mainly the CPW structures. Then, a 3-μm thick sacrificial photoresist layer is spun and patterned to define the anchoring regions of the MEMS devices before a second Al layer is deposited and patterned in the same way as the first one. So, the MEMS beams are defined. Finally, the sacrificial photoresist is ashed to release the devices. The prototype reported in [17], depicted in Figure 4, exhibits a tuning range of 20% and operates at the Q-band. The dimensions of the CSRRs are (in reference to Figure 3) *c* = *d* = 10 μm, *l* = 480 μm and *w* = 130 μm. CPW dimensions are: strip width *W* = 150 μm and slot width *G* = 30 μm. Finally, the geometry of the MEMS bridges is: *B* = 80 μm, *b* = 100 μm, *h* = 40 μm and *H* = 290 μm. The structure is a 4-stage periodic device where the distance between adjacent CSRRs is 220 μm. The simulated (by means of the *Agilent Momentum* by excluding losses) and measured S-parameters of the device are also depicted in Figure 4. As expected, the structure exhibits stop band behavior with tuning capability. The central frequency of the stop band is varied between 39 GHz and 48 GHz for corner actuation voltages of 17 V (down-state) and 0 V (up-state). This corresponds to a tuning range of roughly 20%. Measured rejection in the stop band is good (*IL* > 40 dB), whereas insertion losses in the allowed band are very small.

**Figure 5.**
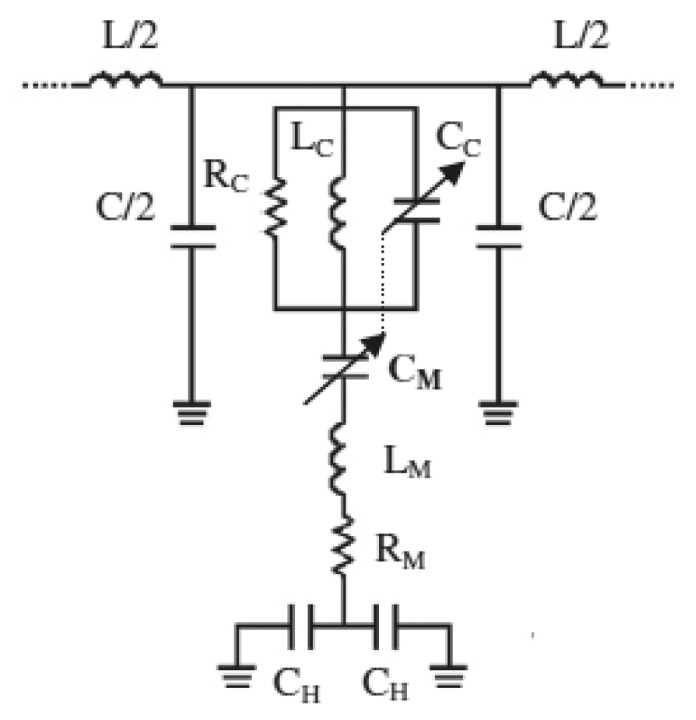
Lumped element equivalent circuit (unit cell) of the CSRR/RF-MEMS based stopband filter, including losses. From [18], reprinted with permission.

The lumped element equivalent circuit model of the unit cell of this CSRR/RF-MEMS based tunable stopband filter was reported in [18], and is depicted in Figure 5. The RF-MEMS bridges are modeled by means of a lumped RLC series circuit, where *C_M_* corresponds to a variable capacitance (having an up-state and a down state value), *L_M_* is the bridge inductance, and the resistor *R_M_* involves the microelectromechanical system losses. The anchoring capacitance of the CPW holes is modeled by *C_H_*. The CPW line is described by means of the per-section inductance, *L*, and capacitance, *C*. The etched CSRRs are modeled by means of a parallel RLC tank, *L_C_* and *C_C_* being the reactive elements which constitute the intrinsic resonance frequency of the resonators, and *R_C_* takes into account the eventual losses associated with the resonator. The intrinsic resonance frequency of the CSRRs is directly affected by the RF-MEMS actuation. In fact, as reported in [18], when *C_M_* is tuned, the electrical properties of CSRRs, including their resonance frequency, are modified. In order to take into account this fact, the capacitor *C_C_* has been represented as dependent on the capacitor *C_M_*. Since the actual RF-MEMS device is anchored directly on the substrate in holes of the CPW ground planes, the anchoring capacitance can be neglected due to its low impact.

**Figure 6.**
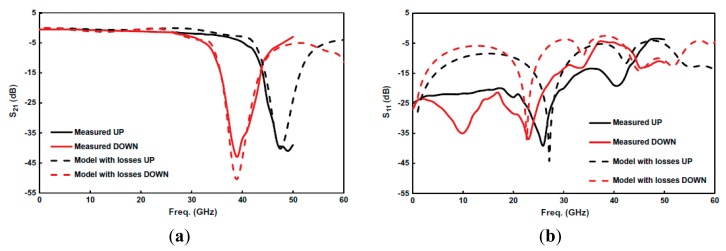
Comparison between the circuit model response (including losses) and the measured response for the designed stop-band reconfigurable CSRR/RF-MEMS based filter. (**a**) Insertion loss; (**b**) return loss. From [18], reprinted with permission.

By considering two different values of the capacitors *C_C_* and *C_M_, i.e.*, those corresponding to the up (*C_CU_* and *C_MU_*) and down (*C_CD_* and *C_MD_*) states, and the other element values fixed, the parameters of the circuit of Figure 5, corresponding to the responses of Figure 4, were extracted in [18]. The following values were obtained: *L* = 0.149 nH, *C* = 59 fF, *L_C_* = 30 pH, *C_CU_* = 0.27 pF, *C_CD_* = 0.41 pF, *R_C_* = 90 Ω, *L_M_* = 38 pF, *C_MU_* = 65 fF, *C_MD_* = 111 fF, *R_M_* = 0.5 Ω and *C_H_* = 0.4 pF. The comparison between the lossy circuit simulations and the measured responses is depicted in Figure 6, where good agreement can be appreciated.

### 2.2. SRR Loaded with Cantilever Type RF-MEMS

**Figure 7.**
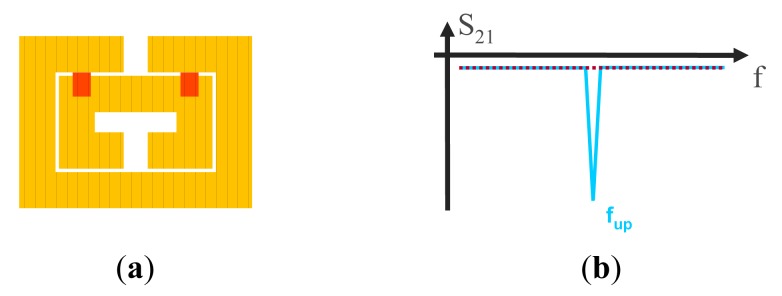
Tunable SRR based on cantilever type RF-MEMS (in red) corresponding to the configuration considered for the implementation of reconfigurable stopband filters (**a**), and sketch of the response of a line loaded with these SRRs for the up (solid line) and down (dotted line) states (**b**).

**Figure 8.**
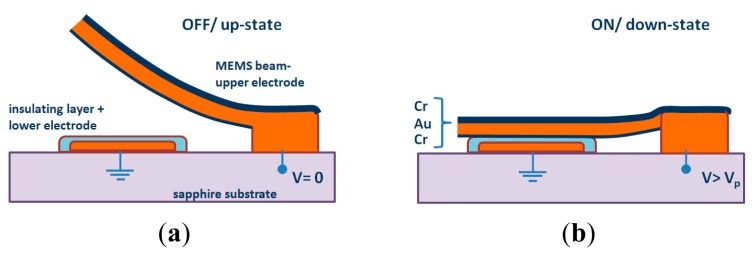
Cross sectional view of a cantilever-type beam RF-MEMS showing the two states: (**a**) non-actuated up-state; (**b**) actuated down-state when the applied voltage is higher than the MEMS pull-down voltage (*V_p_*). From [21], reprinted with permission.

SRR tunability can be achieved by adding cantilever-type RF-MEMS switches, which are composed of one anchor and one movable beam suspended above an actuation electrode. The SRRs and the RF-MEMS can be combined following different configurations, as reported in [21]. The focus here is the configuration depicted in Figure 7, where the external ring is used as DC ground electrode and is the anchor of the cantilever beam, and the internal ring, under them and covered by a thin dielectric layer, acts as the DC actuation electrode (the cross sectional view for the up and down states are depicted in Figure 8). When the cantilever beams are at the up-state, the resulting capacitances formed with the internal ring are low. When they are actuated (down-state), the coupling between rings dramatically increases and this leads to a very large shift of the resonance frequency of the resonator (see Figure 7b).

**Figure 9.**
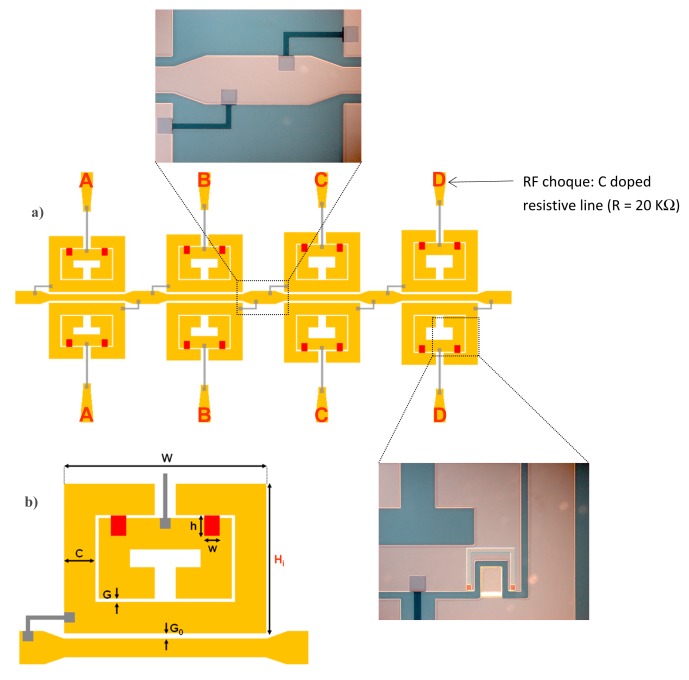
Layout of the four-pole reconfigurable bandstop filter (**a**) and over scale view of one RF-MEMS-loaded SRR (**b**). The total size of the device is 6.4 × 14 mm^2^. The dimensions of the cantilever-type MEMS are *h* × *w* = 200 μm × 150 μm. Width and distance between rings are *C* = 300 μm and *G* = 30 μm. The gap between SRRs and the microstrip line is *G_0_* = 50 μm. The side length of the SRRs in the longitudinal direction is *W* = 1940 μm. Zoom photographs of the indicated parts of the fabricated device are also shown. From [21], reprinted with permission.

**Figure 10.**
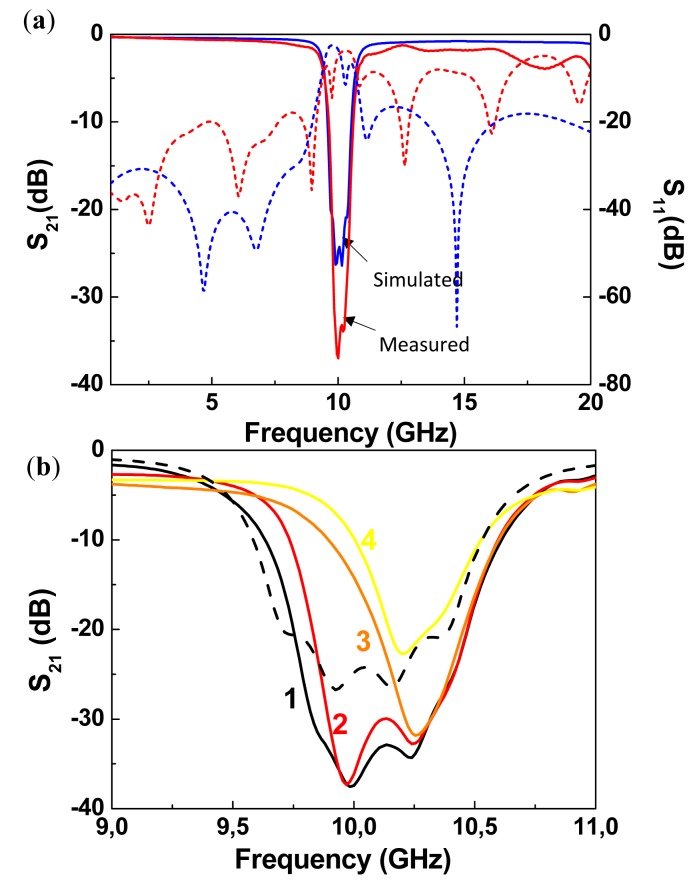
Simulated and measured insertion (solid lines) and return (dashed lines) loss of the 4-pole reconfigurable stopband filter when all switches are at up-state (**a**). Simulated (dashed line) and measured (solid lines) responses of the device for different combinations of switches actuated (**b**). Measurements indicated as 1, 2, 3 and 4 correspond to bits ABCD set to ‘0000’, ‘0001’, ‘0011’ and ‘1011’, respectively, with ‘0’ corresponding to MEMS at up state (*i.e.*, non actuated) and ‘1’ corresponding to MEMS at down state. From [21], reprinted with permission.

Using the configuration depicted in Figure 7a, stopband filters with electronically controllable number of poles can be implemented [21]. By this means, it is possible to tune the filter central frequency and the bandwidth. The idea is to couple multiple resonators, with slightly different resonance frequency, to the host line, following the approach reported in [11]. If the resonators are uncoupled, each resonator contributes with a filter pole (transmission zero) and bandwidth can be tailored. In the framework of this approach, it is clear that filter characteristics can be tuned by removing one or more resonators (and hence the corresponding poles). However, by using MEMS switches in combination with SRRs according to the configuration of Figure 7a, the poles can be removed without the need for resonator removal. We simply need to actuate the MEMS, and the pole (or poles) of the corresponding SRR will be largely shifted. Following this idea, a four-pole reconfigurable bandstop filter, consisting of a 50 Ω microstrip transmission line loaded with four pairs of RF-MEMS-loaded SRRs, was designed and fabricated (Figure 9a) [21]. The difference between SRRs called A, B, C and D is the side length *H_i_* of the external ring (Figure 9b), where *H_A_* = 1430 μm, *H_B_* = 1475 μm, *H_C_* = 1530 μm and *H_D_* = 1580 μm. Without electrostatic actuation, this configuration provides a bandstop behavior with four poles corresponding to the resonance frequencies *f_A_* (10.36 GHz), *f_B_* (10.15 GHz), *f_C_* (9.92 GHz) and *f_D_* (9.73 GHz) of the SRRs of cells A, B, C and D, respectively (Figure 10). As shown in Figure 9a and b, the common DC ground signal is supplied to all external rings through the transmission line and resistive lines, while each internal ring acts as a DC independent electrode.

The reconfigurable filter, designed to operate at the X-band, was designed with ON/OFF RF-MEMS switches to provide a ratio between up-state and down-state capacitances of 10, which leads to a shift of the resonance frequencies of the resonators from X-band to L-band. Owing to the actuation of switches and taking into account that both SRRs of one cell must always present the same resonance frequency, we obtain a 4-bit (called A, B, C and D) reconfigurable filter, where we can digitally tune the filter bandwidth and central frequency. The measured insertion and return losses of the filter with all MEMS at up-state (non-actuated) are presented in Figure 10a and compared with the full wave simulations. The filter exhibits a four pole rejection band around 10 GHz and the rejection is higher than 20 dB on a 0.72 GHz frequency range. There is good agreement between simulation and experiment, except that out of the stop band measured insertion losses are higher and return losses are lower than those predicted by the simulation. This is due to the connection between the transmission line of the filter and the two SMA connectors. Other measured filter responses corresponding to different combinations of switches simultaneously actuated with 60 V are depicted in Figure 10b. The number of poles of the stop band corresponds to the number of non-actuated switches. With these results, the digital reconfigurability principle is validated.

Concerning fabrication, the actuation electrodes were realized by the thermal evaporation of a Cr/Au (60/1200 Å) thin layer on a 250-μm thick Sapphire substrate (with dielectric constant *ε_r_* = 9.8). They were covered by a 0.4-μm thick Al_2_O_3_ dielectric layer deposited by plasma-enhanced chemical vapor deposition (PECVD). The alumina dielectric layer serves as electrical insulator between the lower electrode and the MEMS cantilever beam (the upper moveable electrode, as shown in Figure 8). It follows the lift-off of a 50-nm thick doped Carbon layer, deposited by reactive laser ablation, to realize the 20 KΩ resistive lines (see Figure 9). The suspended parts of the structure (moveable cantilever beam) were defined by patterning a 0.5-μm thick sacrificial polymethylglutarimide (PMGI) resist. The metallization was done using the Cr/Au seed layer which is gold-electroplated up to 1.5 μm. Next, a 90 Å Cr stress layer was deposited and patterned, in order to provide an appropriate stress gradient in the foldable areas. Finally, the device was realized and dried in a critical point drying system to avoid it sticking to the dielectric of the suspended structures. As illustrated in Figure 9, the structure integrates carbon-doped resistive lines and metallic polarization pads for the electrostatic actuation of the RF-MEMS switches.

## 3. Stopband Filters Based on Cantilever Type SRRs

**Figure 11.**
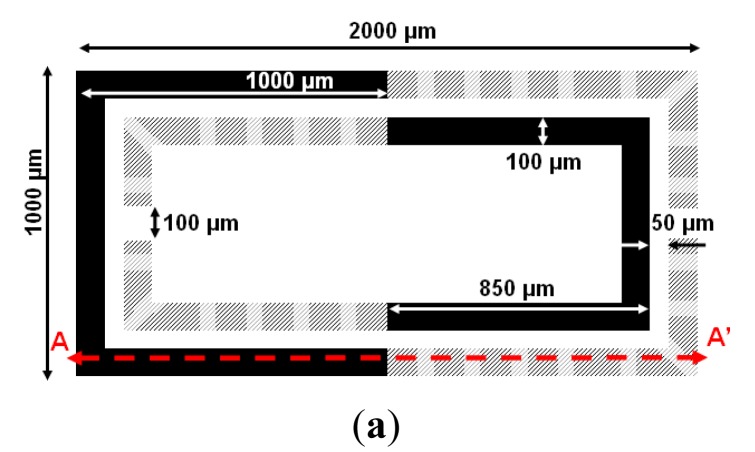

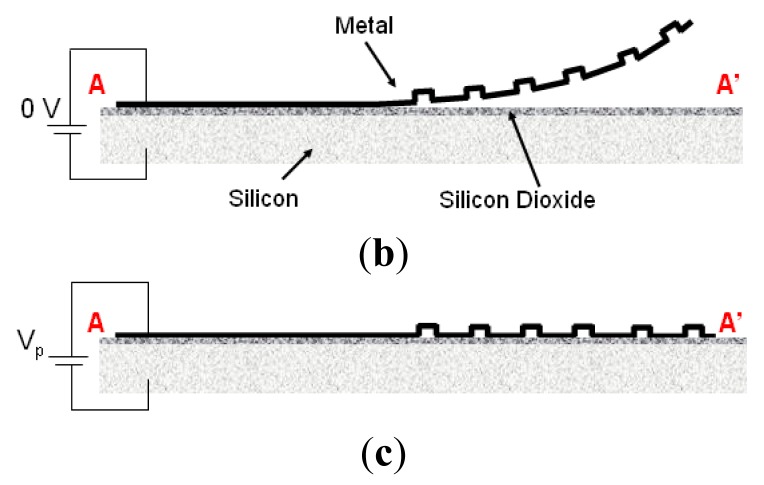
Tunable SRR based on cantilever-type RF-MEMS. (**a**) Top view with relevant dimensions. Black and grey parts correspond to anchors and suspended parts (including corrugations), respectively; (**b**) cross section in the up state; (**c**) cross section in the down state. The 500-μm thick high resistivity silicon (HR-Si) substrate is electrically isolated from the anchor through a 1-μm thick SiO_2_ layer. From [22], reprinted with permission.

An alternative approach for the implementation of tunable resonators consists of using the RF-MEMS as part of the SRR [22]. The rings forming the SRRs are partly fixed to the substrate (anchor) and partly suspended (up-curved cantilever). Through electrostatic actuation, the suspended parts are deflected down, the distributed capacitance between the pair of coupled rings is modified, and hence the resonance frequency of the SRR can be electrically tuned. A typical top view of the cantilever type tunable SRR (rectangular shaped), is depicted in Figure 11a. The movable parts of the rings are indicated in grey. Obviously, we can arbitrarily select the movable portion of each ring, which has direct influence on the tuning range. Figure 11b and c depicts the cross-sectional view of the anchor and the cantilever, without (up state) and with (down state) electrostatic actuation, respectively. The fabrication process is similar to that explained in the previous section.

**Figure 12.**
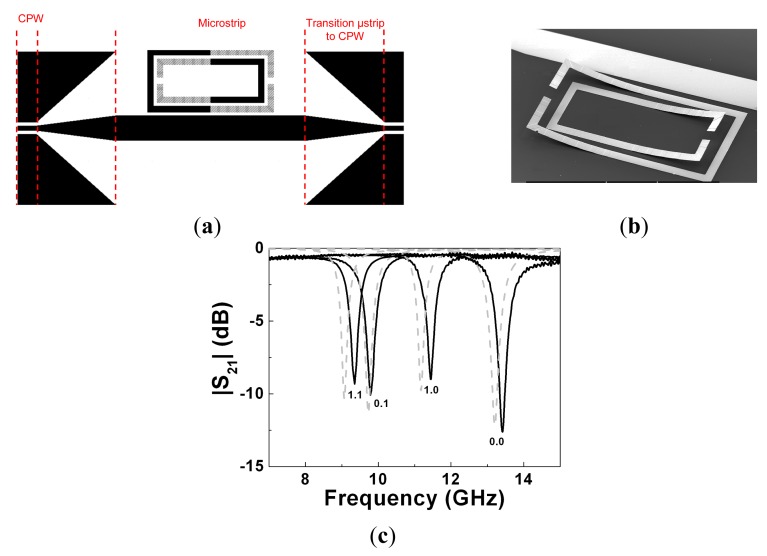
Topology of the tunable SRR coupled to a microstrip line with microstrip to coplanar waveguide transition (**a**), photograph of the non-actuated SRR (**b**), and measured (solid lines) and simulated (dashed lines) frequency response of the structure for the four different states (**c**). The separation between the SRR and the microstrip line is 50 μm, and the width of the microstrip line is 400 μm. The applied voltage for either ring actuation is 30 V. The state of the rings is indicated, where ‘1’ (ring actuation) stands for down state and ‘0’ for up state, and the first bit corresponds to the inner ring. From [22], reprinted with permission.

The tuning principle was validated in [22] by coupling the SRR of Figure 11 to a 50 Ω microstrip line (Figure 12). Since we can independently actuate on both the internal and external ring of the tunable SRR, four different states arise. The measured transmission coefficients corresponding to the four states are also depicted in Figure 12. Without actuation (both cantilevers at up-state), the resonance frequency of the SRR is 13.42 GHz. It decreases to 11.45 GHz by actuating the outer ring or to 9.78 GHz by actuating the inner ring. The smaller resonance frequency (9.34 GHz) is that corresponding to the two rings in the down state, as expected on account of the larger distributed capacitance between the two rings of the SRR. The experimental responses are in good agreement with the simulated ones. For the simulation, either ring in the up state was modeled as composed of two parts: (i) a portion accounting for the anchor and thus in contact with the SiO_2_ layer; and (ii) an elevated portion, with an uniform and effective height (*h_eff_*) from the SiO_2_ layer, corresponding to the movable part, in contact with the anchor by means of a metallic via. In this model, the effects of rings corrugation were neglected, and the distributed capacitance between the rings in the up state was approximated by the capacitance between non-coplanar parallel strips separated a vertical distance *h_eff_*. The electromagnetic simulations of the structure, modeled as reported above, were carried out by means of the commercial software *Agilent Momentum* (considering *h_eff_* as an adjustable parameter). Good agreement between measurement and simulation for the four states was obtained by choosing *h_eff_* = 17 μm. This effective height is substantially smaller than the actual (maximum) elevation of the rings in the up state, which was estimated to be roughly 100 μm. However, this is expected since the per-unit length capacitance of the pair of rings decreases dramatically when their separation increases.

**Figure 13.**
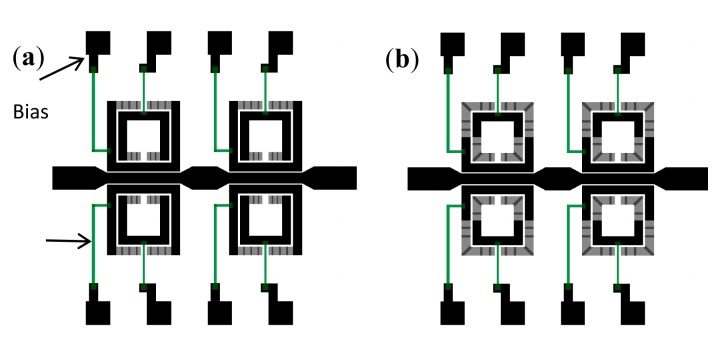

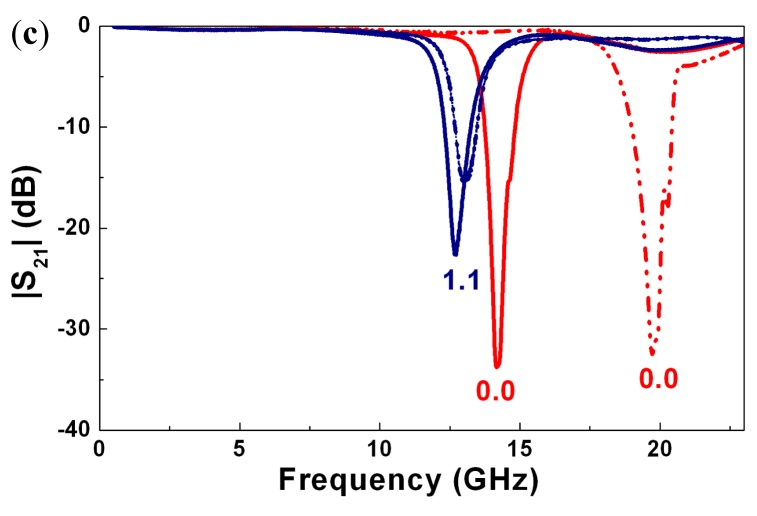
Tunable stopband filters based on square-shaped short (**a**) and long (**b**) cantilever-type SRRs, and measured transmission coefficients for the extreme switching states (**c**). SRR side length is 1200 μm, ring width 150 μm and ring separation 30 μm. The separation between the SRR and the microstrip line is 25 μm. The actuation voltages are applied to the rings through the bias pads and high resistive lines (HRLs). Solid lines correspond to the filter of (a); dash-dotted lines correspond to the filter of (b). Actuation voltage is 30 V. From [22], reprinted with permission.

By cascading the proposed MEMS-based SRRs in a microstrip transmission line, tunable stopband filters can be implemented (the rejection level can be controlled by the number of stages). Two prototype devices are depicted in Figure 13, where the difference between them is simply the length of the movable portions of the rings. The measured frequency responses corresponding to the extreme switching states (‘00’ and ‘11’) are also depicted in Figure 13. The tuning range is 12% for the filter of Figure 13a and 42% for the one depicted in Figure 13b. This difference is due to the larger capacitance variations experienced with the prototype that uses longer cantilevers. As compared to tunable stopband filters based on SRRs and varactor diodes, the filters of Figure 13 exhibit better insertion losses in the allowed bands. As compared to the filters based on tunable RF-MEMS based CSRRs of Figure 4, the approach presented in this Subsection, based on cantilever type SRRs, can provide better tuning ranges.

## 4. Discussion and Comparison

The main relevant advantages of the proposed reconfigurable filters, as compared to stopband filters based on SRR or CSRR loaded with varactor diodes [12,14], are operation at higher frequencies and lower level of insertion losses in the allowed bands. As compared to stopband filters based on SRRs combined with ferroelectric materials [15], the tuning voltages required in RF-MEMS based filters are typically smaller, and losses are less significant as well. Notice that the filters reported in Figures 4 and 13 are similar in the sense that the resonance frequency of the SRRs (all identical) is modified by means of MEMS actuation. However, the tuning range is superior in the filter of Figure 13 since the allowable variations of resonator capacitance are larger in cantilever type SRR, as compared to CSRRs loaded with bridge type RF-MEMS. The filter of Figure 9, based on a different principle (digital tuning), is specifically useful to achieve independent control of central frequency and bandwidth. Other tunable stopband filters and bandpass filters based on combinations of split rings and RF-MEMS are reported in [21]. It is also worth mentioning that the high sensitivity of the resonance frequency (or capacitance) of the cantilever type SRR in the filter of Figure 13b with cantilever deflection, points out that these structures can potentially be of interest for the implementation of position or pressure sensors.

## 5. Conclusions

In conclusion, we have reviewed three strategies for the implementation of reconfigurable stopband filters based on the combination of split ring resonators (SRRs), or their complementary counterparts (CSRRs), with RF-MEMS switches. In two of the considered approaches, the SRRs/CSRRs are loaded with cantilever/bridge type RF-MEMS, whereas in the third approach, the cantilever RF-MEMS are part of the SRRs. Several prototype devices have been reported as proof-of-concept. Small loss level, operation at high frequencies, high tuning ranges and/or the possibility to independently control the central frequency and bandwidth are characteristics that can be achieved by the different considered configurations.
